# Work and children in Spain: challenges and opportunities for equality between men and women

**DOI:** 10.1007/s13209-021-00243-7

**Published:** 2021-10-04

**Authors:** Claudia Hupkau, Jenifer Ruiz-Valenzuela

**Affiliations:** 1 Department of Economics, CUNEF Universidad, Madrid, Spain; 2 Department of Economics, Universitat de Barcelona & Barcelona Institute of Economics (IEB), Barcelona, Spain; 3 Centre for Economic Performance, London School of Economics, London, UK

**Keywords:** Gender gaps, Inequality, Family policy, Motherhood penalty, J13, J16, J18

## Abstract

Over the past decades, Spain has seen a striking convergence between women’s and men’s participation in the labour market. However, this convergence has stalled since the early 2010s. We show that women still fare worse in several important labour market dimensions. Gender inequalities are further aggravated among people with children. Women with children under 16 are much more likely to be unemployed, work part-time or on temporary contracts than men with children of the same age. We show that it is unlikely that preferences alone can account for these gaps. A review of the evidence shows that family policies, such as paternity leave expansions, financial incentives in the form of tax credits for working mothers and subsidised or free childcare for very young children, could help reduce the motherhood penalty. However, such policies are likely to be more effective if combined with advances in breaking up traditional gender roles.

## Introduction

**Fig. 1:**
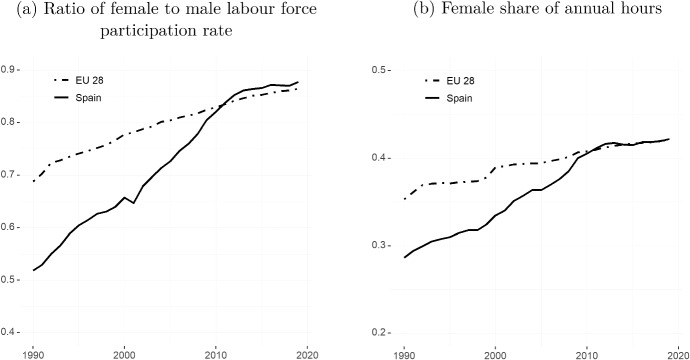
Trends in female employment and hours worked Source: OECD Labour Force Statistics. The left-hand side graph **a** shows the female to male labour force participation (LFP) rate. A value of one indicates that the female LFP rate is equal to that of men. A value below (above) one indicates that the female LFP rate is below (above) that of men. The LFP rate is defined as the share of the working age population (15–64) that is either employed or unemployed. The right-hand side graph **b** shows the female share of total annual hours worked among all workers. Total annual hours worked have been computed as the average usual weekly hours worked on the main job multiplied by the total number of employed individuals

Over the past 25 years, Spain has undergone a striking convergence between women’s and men’s participation in the labour market. In 1990, for every 100 men in the labour force there were only 50 women working, compared to 70 women for every 100 men in Europe overall. By 2010, Spanish women’s labour market participation had overtaken that of women in the European Union, with about 88 active women for every 100 active males, compared to 86 in the EU overall (Fig. 1a). Women have also drastically increased their share of total hours worked, from 29 percent in 1990 to 42 percent in 2019, as can be seen in Fig. 1b. However, as in other developed countries, gender convergence in Spain has stalled since the early 2010s.

In this paper, we use data from the Spanish Labour Force Survey (Encuesta de Población Activa, or EPA) and the Living Conditions Survey (Encuesta de Condiciones de Vida, or ECV) to study the evolution of gender gaps in several key labour market indicators over the past 15 years. We then analyse these gaps separately for individuals with and without children, to get a better understanding of the extent to which children might matter for explaining why convergence has stalled in recent times.^1^ In light of the Covid-19-related recession, we separately investigate the impact of the pandemic on gender inequality in labour market outcomes in Spain. Finally, we review the empirical evidence on policies that might help reduce the gender gap associated with the arrival of children.

Our results show that despite convergence between men and women in participation rates, women still fare worse on other important labour market indicators. With the exception of a period after the Great Recession, which hit male-dominated sectors disproportionately, unemployment among women remained several points higher than that of men throughout the whole period. Despite the increase in working hours seen in Fig. 1b, the proportion of women working part-time has barely changed in the last 15 years and remains well above 20 percent. Men’s part-time employment share has stayed below 10 percent throughout the period. Women are also more likely to hold job-insecure contracts. Moreover, only about 3 percent of women in Spain work in top-level occupations, such as directors and managers—about half the fraction of men. This is despite the fact that the share of men in such occupations has been decreasing steadily over the last decade.

Labour market inequalities in Spain are further aggravated among people with children, irrespective of the indicator used. We find that convergence in labour force participation rates has stagnated for women with children aged 15 and below over the last seven years, while convergence has continued (albeit at a slower pace than before) among individuals without children. By 2019, women with children under 16 years of age were about 7.5 times more likely than men with children of the same age to work part-time, twice as likely to be unemployed and 20 percent more likely to hold temporary contracts (as opposed to a more job-secure, permanent contract). The gender gaps for people without children in all these indicators are much smaller. We show that it is unlikely that these differences in part-time work uptake are due to women’s preferences alone: by the end of the 2010s, over a third of women with children under five and working part-time stated that they would like to work more hours. This increases to well over half of the women working part-time with children aged five to 15.

Additionally, we show that substantial gender gaps in gross annual income exist, but only among people with children. Using the Living Conditions Survey for the period spanning 2008 to 2019, we find that among full-time workers, women with children aged zero to 15 have a gross annual income of between 78 and 87 percent of that of men. This is consistent with results reported in de Quinto et al. (2020), who find that mothers’ earnings in Spain drop by 11 percent one year after childbirth and by 28 percent after ten years, while those of fathers remain unchanged.

We also find that the gender gap in the labour market effect of the Covid-19 crisis is larger among people with children. Drops in employment rates were three percentage points higher for mothers than for fathers by the fourth quarter of 2020, and mothers were more likely to have moved into inactivity during the pandemic. Among people without children, we do not find gender differences in the impact of the pandemic on these outcomes. However, in line with recent findings by Bluedorn et al. (2021), we show that the gender gap among people with children had largely been reversed by the first quarter of 2021. It remains to be seen whether the temporary labour market shocks for mothers will have longer-lasting impacts on their careers and on the division of labour in the household.

The fact that the arrival of children seems to be one of the main drivers of the remaining gender gap in labour market outcomes suggests that the focus of policy makers should lie in policies that reduce the unequal impact of childbearing on the careers of men and women. Our review of the existing evidence suggests that policies that make it easier to combine work and family for women, such as financial incentives in the form of tax credits for working mothers and subsidized or free childcare for very young children, can positively affect women’s employment probabilities and hours worked (Olivetti and Petrongolo 2017). These latter policies have also been shown to be effective in tackling a related issue: steadily declining fertility rates in developed countries. However, such policies are likely to be more effective if combined with advances in breaking up traditional gender roles.

Our paper updates and extends the existing literature on gender gaps in the labour market for the case of Spain. The analysis is most closely related to Guner et al. (2014), who analyse the evolution of gender gaps in Spain from the late 1970s to 2013. We increase the time horizon of analysis up to 2020 and pay special attention to (1) the differences in the gender gaps between individuals with and without children and (2) the effect of the Covid-19 pandemic on existing labour market gender gaps. Additionally, the review of the literature on family policies aims to contribute to the debate on what policies work to reduce gender gaps in the labour market.

**Fig. 2:**
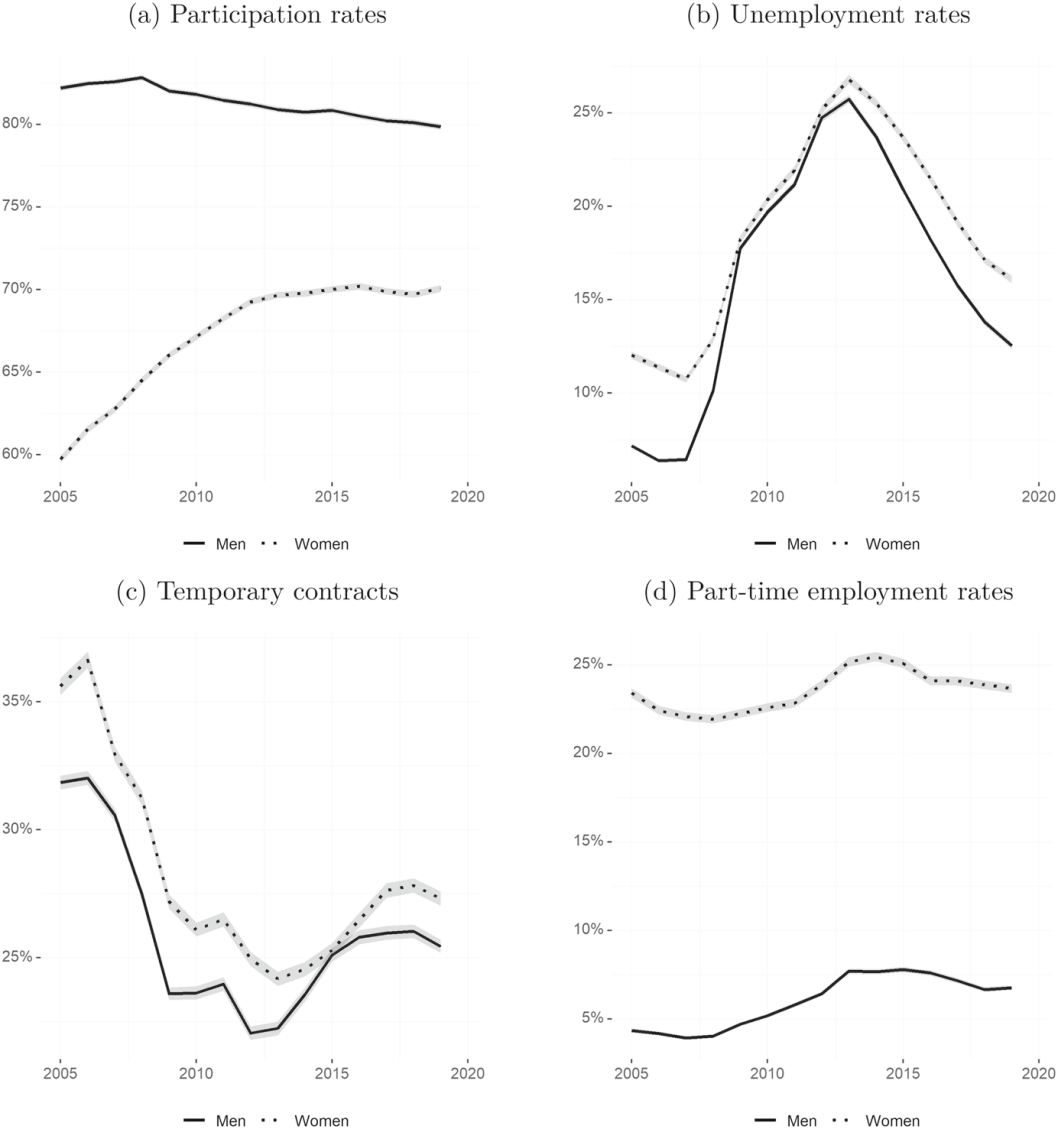
Labour market outcomes for men and women (2005–2019) Source: Own calculations based on EPA microdata. Seasonally adjusted series from Q1/2005 to Q4/2019. Sample of all individuals within the working-age population (16–64 years). Participation rates are computed as the total active population (employed and unemployed) over the total working-age population. Unemployment rates are computed as the total number of unemployed over the total active population. Temporary contracts show the share of individuals with a temporary contract among all those in employment. Part-time employment rates show the share of individuals working part-time among those in employment. All variables are derived using cross-sectional weights. Shaded areas represent 95% confidence intervals

The remainder of this paper is organised as follows. In Sect. 2 we review gender gaps in the Spanish labour market over the past 15 years. Section 3 looks more in detail at how parenthood affects labour market outcomes for Spanish men and women. Section 4 reviews the existing evidence on family policies and their impact on female employment and fertility. Section 5 concludes.

## Gender gaps in the Spanish labour market over the past decades

In this section we document the evolution of several main labour market indicators for men and women in the working age population in Spain. Figure 2 plots, separately for men and women, the participation and unemployment rates, as well as the fraction of men and women employed in part-time and temporary contracts from 2005 to 2019.^2^

The increase in women’s participation rates initiated several decades ago continued until the early 2010s, when almost 70 percent of working age women were active in the labour market. However, the participation rate has since stalled. If the female to male ratio in participation rates shown in Fig. 1a still showed a slight convergence during the last seven years, it is because the male participation rate has suffered a decrease from the early 2010s (Fig. 2a).

The female unemployment rate stood at 13 percent in 2005, about 5 percentage points higher than the male counterpart. The Great Recession years in Spain, with a high volume of employment destruction in male-dominated industries such as construction, brought the gender gap in unemployment rates to almost zero. Both the female and male unemployment rates increased dramatically and reached 25 percent in 2013. However, as the economy started its recovery, the male unemployment rate decreased at a faster pace. This chimes well with the analysis of Dolado et al. (2020)), who examine changes in workforce composition during the Great Recession. They find that those changes were reversed during the subsequent recovery phase, confirming their temporary nature. In the Spanish case, by the end of 2019, the female unemployment rate had decreased at a slower rhythm than that of males and was about 16 percent (i.e. 3 percentage points) higher than the male analogue (Fig. 2b).

Employed women have been more likely to hold job-insecure contracts than men throughout the last fifteen years, with the exception of a very brief period during the economic recovery of 2014 and 2015, when the gender gap in temporary employment rates vanished. After that, the gender gap has increased again. By the end of 2019, about 27 percent of women and 25 percent of men were employed under fixed-term contracts (Fig. 2c).

However, the biggest gender gap among the four indicators depicted in Fig. 2 is seen in the fraction of workers employed part-time. Whereas well above 20 percent of employed females worked part-time throughout the period, the male part-time rate never reached 10 percent. Overall, the gender gap in part-time contracts has remained stable (Fig. 2d). As we will see in the next section, it is unlikely that this is solely due to differences in preferences for shorter working hours between men and women.

**Fig. 3:**
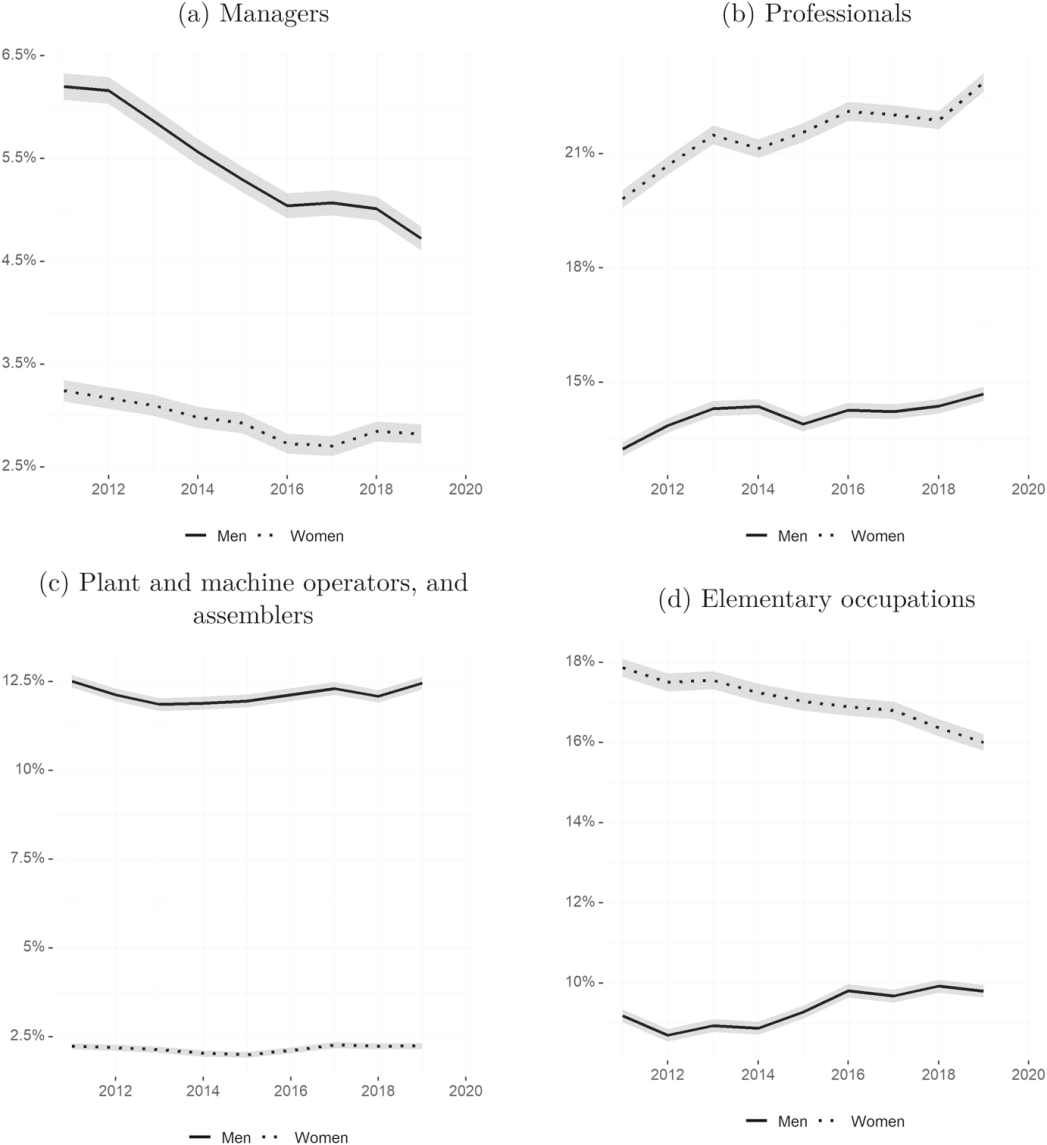
Percent employed in top and bottom occupations. Source: Own calculations based on EPA microdata. Seasonally adjusted series from Q1/2011 to Q4/2019. Variables show the share of individuals working in the specified CNO-11 occupations over the total number of employed individuals, by gender. All variables are derived using cross-sectional weights. Shaded areas represent 95% confidence intervals

In Fig. 3 we turn our attention to the evolution of the fraction of males and females employed in the top two occupations (managers and professionals, respectively) and bottom two occupations (plant and machine operators, and elementary occupations, respectively).^3^ Slightly more than 3 percent of employed females were working as managers at the beginning of the 2010s, while this percentage was between 4.5 and six for men throughout the whole period. In fact, the fraction of women employed in the top occupation fell to below 3 percent from 2015 onward. Indicators of the gender gap for this outcome could be misleading: This is because the fraction of males working as managers has suffered a steady decline since the early 2010s. On the flip side, the fraction of both males and females employed as professionals (e.g. engineers, teachers, doctors or lawyers) has risen in the last decade, and the rise has been bigger for females. This is the only indicator analysed here where women consistently fare better than men. The large gender gap in favour of females has been widening throughout the last decade.

In the bottom two occupations, relatively more men than women work as plant and machine operators. The gender gap is about 10 percentage points (around 12.5 percent versus 2.5 percent) in favour of men and has remained stable over the last decade. The situation is reversed for elementary occupations (including cleaners, domestic workers, waste collectors and food preparation workers), with 18 percent of working women sorting into this occupational group in 2011, compared to about 9 percent of men. The gap in favour of women has decreased, mainly due to a smaller fraction of women working in that occupation (about 16 percent), at the end of the period.

The stagnation in gender convergence found in Spain over the past decade closely mirrors patterns observed in other industrialised economies. While many have seen a steady convergence in labour market outcomes, especially after the 1950s, this has been followed by a plateau in female participation rates starting around the 1990s in Denmark and Sweden, and in the 2000s in the USA, the UK and Norway (Olivetti and Petrongolo 2016). This has raised the question of what the drivers for the persistence in gender gaps are, and there is emerging consensus that the arrival of children plays a major role.

## Parenthood and the Spanish labour market

Several recent studies show that differences in both the labour market attachment and earnings between men and women appear and amplify after entry into motherhood. For instance, using Danish administrative data, Kleven et al. (2019) show that the arrival of children creates a long-run gender gap in earnings of around 20 percent that is driven by hours worked, participation and wage rates. In Spain, de Quinto et al. (2020) find that mothers’ earnings drop by 11 percent, while those of fathers remain unchanged one year after child birth, and this drop rises to 28 percent for women ten years after giving birth. They also show that the drop seems to be driven by mothers shifting into part-time work and temporary contracts. Using data from 29 European countries, Berniell et al. (2020) show that the arrival of the first child is associated with a sharp drop in employment and an increase in part-time work and self-employment for mothers, while it has no effect on these outcomes for fathers.

Following this literature, this section provides an overview of how gender gaps in the main indicators described earlier have evolved over the last 15 years for people with children aged zero to 15 and people without children. We concentrate on males and females aged 25 to 54 to avoid capturing gender differences in education participation when young, and retirement at older ages.

**Fig. 4:**
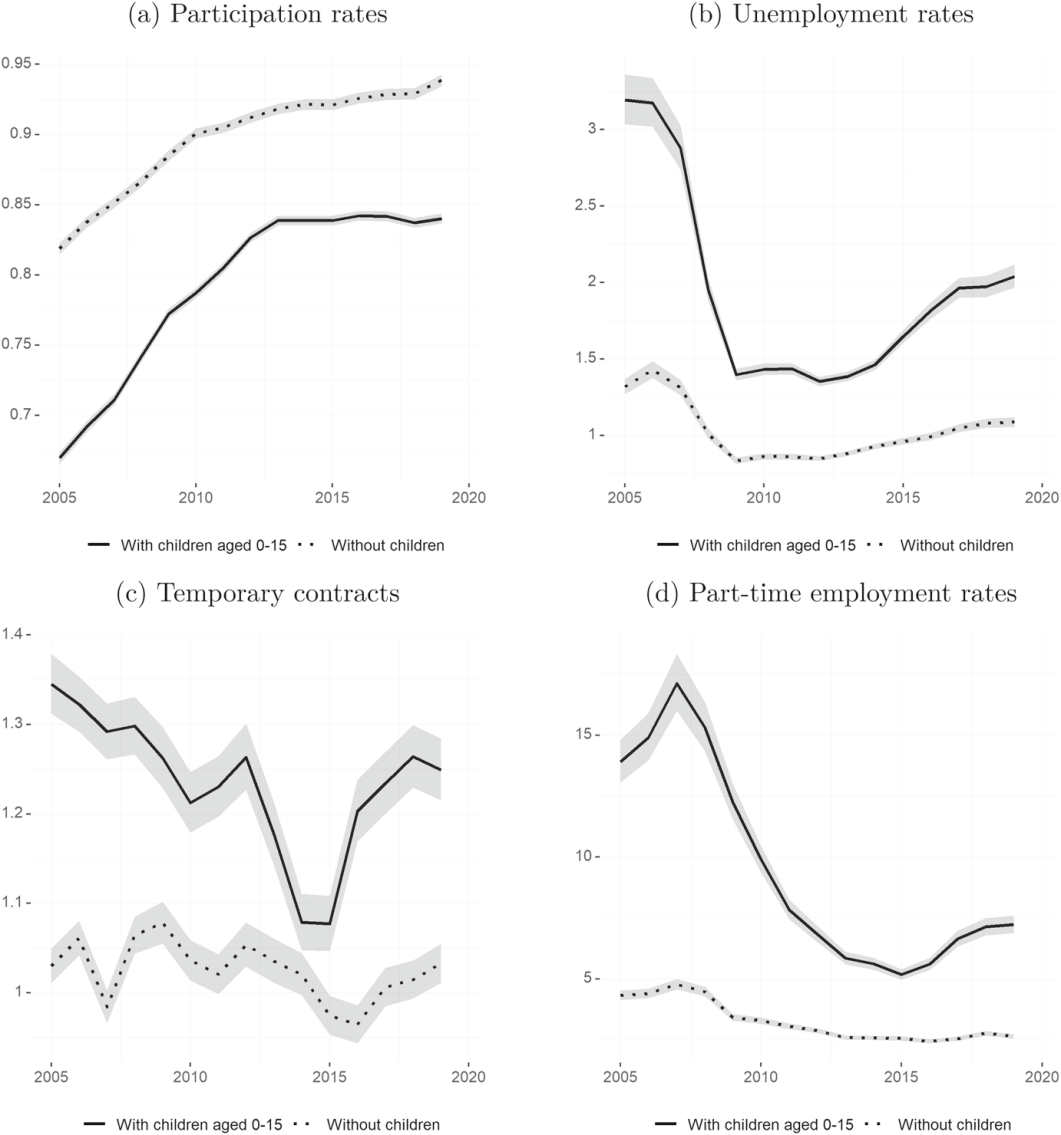
Female/male ratios in labour market outcomes—by whether has children (2005–2019) Source: Own calculations based on EPA microdata. Seasonally adjusted series from Q1/2005 to Q4/2019. Sample of individuals aged 25–54. Plotted series are computed as the ratio of the rate for women over the rate for men. Participation rates are computed as the total active population over total working-age population. Unemployment rates are computed as the total number of unemployed over the total active population. Temporary contracts show the share of individuals with a temporary contract among all those in employment. Part-time employment rates show the share of individuals working part-time among those in employment. All variables are derived using cross-sectional weights. Shaded areas represent 95% confidence intervals

In Fig. 4, rather than expressing gender gaps as the difference between male and female rates, we plot the ratio of female over male rates. Thus, the *y*-axis scale shows how many times more likely women are to be in a particular situation than men. The picture that emerges is clear and similar across indicators. First, whereas women without children have almost converged in terms of participation in the labour market (the ratio of the female to male participation rate for this group is close to one), women with children under 16 are still considerably less likely to participate than men: By the end of the 2010s, only about 85 women with children work for every 100 men with children that do so (see Fig. 4a).

The situation is similar when it comes to unemployment rates, shown in Fig. 4b. The ratio of female to male unemployment rates for those without children fluctuates around one, implying very small gender differences. In contrast, women with children aged under 16 were about 3 times more likely to be unemployed than their male counterparts at the beginning of the period under analysis. The Great Recession drove the ratio down to just above 1.5, but it started increasing as economic activity picked up. By the end of the 2010s, women with children were two times more likely to be unemployed than similar men.

Since the beginning of the 2010s, women without children are about ten percent more likely to be employed under more job-insecure contracts—temporary or fixed-term contracts—than men without children (Fig. 4c). This gap, once more, is larger for women with children aged 15 or under. With the exception of a brief period around 2015, women with children have been 20 to 30 percent more likely to be employed under temporary contracts than men with children.

**Fig. 5:**
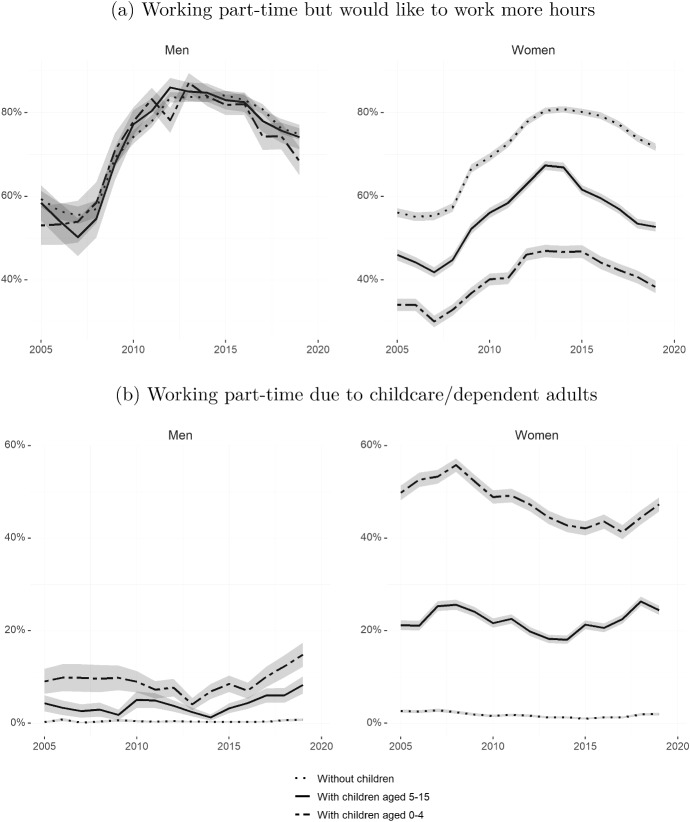
Preferences and reasons for part-time employment (2005–2019) Source: Own calculations based on EPA microdata. Sample of individuals aged 25–54 working part-time. The left-hand side graph **a** shows the share of individuals who would like to work more hours among those in part-time employment. The right-hand side graph **b** shows the share of individuals who state childcare or caring for dependent, ill, or aged adults as the reason for working part-time. All variables are derived using cross-sectional weights. Shaded areas represent 95% confidence intervals

The starkest differences come with the ratio of female to male part-time rates, shown in Fig. 4d. Women without children are about 2.5 times more likely to work part-time. For people with children, the ratio increases to around 15 between 2005 and 2010, after which it decreases to its minimum in 2015. By 2019, women with children are about 7.5 times more likely to be working part-time than similar men.

It is unlikely that these differences in part-time work are driven by women’s preferences for shorter hours alone. In Fig. 5a we plot the fraction of men (left) and women (right) that are working part-time but would like to work more hours. Among individuals without children working part-time, men and women are equally likely to state that they would like to work more hours throughout the whole period of analysis. By 2019, this fraction stood at 74 percent for women and 76 percent for men.

While there are no significant differences in the preferences to work more hours for men with and without children, for women, the presence of children matters a lot. About 50 to 60 percent of mothers with children aged five to 15 who are working part-time would like to work more hours, and this percentage is still a sizeable 30 to 40 percent for those with children aged zero to four.

When asked directly about the reasons to work part-time, children and care responsibilities are stated by about 40 to 55 percent of women with children under five and by about 20 percent of women with children aged five to 15 (see Fig. 5b). These rates are much lower for fathers—around ten percent for those with children aged five to 15, and fluctuating around 15 percent for those with children aged zero to four. As before, in Fig. 5a, a clear hierarchy by child age is observed for women, and a much less pronounced hierarchy is appearing for men by the end of the period.

**Fig. 6:**
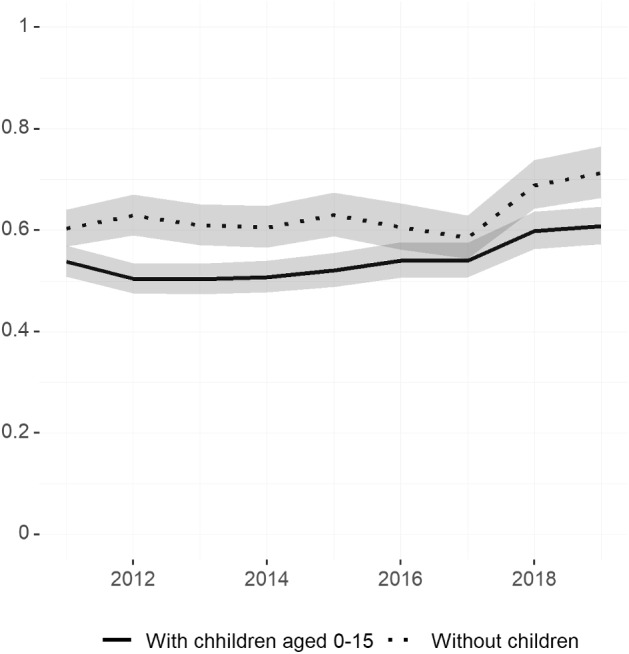
Female/male ratios of percent working as directors and managers—by whether has children (2011–2019) Source: Own calculations based on EPA microdata. Seasonally adjusted series from Q1/2011 to Q4/2019. Sample of individuals aged 25–54. Variables show the ratio of the share of women working in the CNO-11 occupations classified as directors and managers over the share of men working in this occupational category. The share of individuals working in this CNO-11 occupation is computed over the total number of individuals in work. All variables are derived using cross-sectional weights. Shaded areas represent 95% confidence intervals

We now turn to inequalities in occupational choices and whether parenthood is relevant for explaining part of the gender gap in this dimension. Figure 6 shows the ratio of females over males employed in top-level occupations (directors and managers) for those with children aged zero to 15 and for those without children of this age. In both cases, women are less likely to be observed in the top managerial positions. By the end of the 2010s, women without children were about 30 percent less likely than men without children to be employed as managers. This gap increases to about 50 percent for women with children aged zero to 15 for most of the period, with slight improvements over the last two years.

Another well-studied dimension of labour market gender gaps is that of earnings (or income). Having seen the higher rate of part-time work among females overall, we use data from the Spanish Living Conditions Survey from 2008 to 2019 for individuals aged 25 to 54 to understand the evolution of income gender gaps for full-time workers only.^4^

**Fig. 7:**
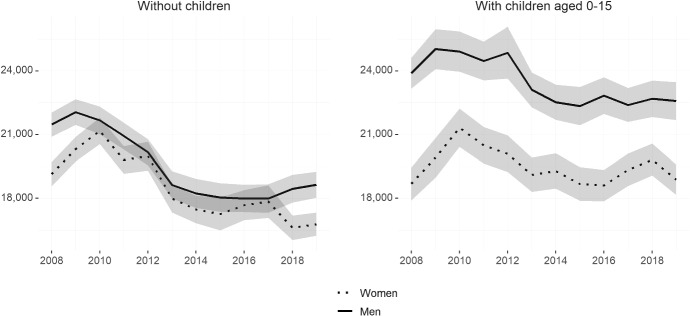
Annual gross income of full-time workers—by gender and whether has children (2008–2019) Source: Own calculations based on ECV microdata from 2008–2019. Sample of individuals aged 25–54. Variables show the cpi-deflated annual gross income by whether has children for men and women. All variables are derived using cross-sectional weights. Shaded areas represent 95% confidence intervals

Figure 7 shows annual gross income for full-time workers without children (left) and with children aged 15 and under (right). Similar to what we have seen with other labour market indicators, in the aftermath of the Great Recession annual gross income is not significantly different between men and women without children. However, both before 2010 and after 2017, women without children working full-time have an annual gross income close to 2000 euros lower than men. Following the pattern seen for other indicators, the gender gap in annual gross income among people with children aged below 16 is bigger throughout the period, fluctuating between 3000 and 5000 euros in favour of men. That is, women with children aged zero to 15 working full-time have an annual gross income of between 78 and 87 percent of that of men with children of the same age. The literature has pointed toward several factors that could explain the earnings penalty for women with children, even when they keep on working full-time. These include the lower likelihood of receiving promotions (Kleven et al. 2019) and the higher likelihood of switching to occupations with more flexible working hours and where workers are more easily substituted (Azmat et al. 2021).

All in all, and despite presenting just descriptive evidence, the picture that emerges mirrors findings from other studies offering causal evidence: There seems to be a child penalty for women in Spain when it comes to labour market outcomes as measured by the participation rate, job insecurity, hours worked, employment in top occupations and income (even for full-time workers).

### The Covid-19 pandemic and gender gaps

The year 2020 was marked by one of the severest recessions in the last century as a result of the Covid-19 pandemic. Spain has been hit exceptionally hard, both in terms of its human as well as its economic toll.^5^ Due to the nature of the crisis, where jobs in female-dominated sectors (e.g. service industry) were disproportionately affected by prolonged shut-downs, some studies have shown that women were affected more severely than men by job and earnings losses initially (Adams-Prassl et al. 2020; Alon et al. 2020). For Spain, Hupkau and Victoria (2020) show that women were more likely to be working in sectors that were affected by shut-downs and Farré et al. (2020) find that women were slightly more likely overall to have lost their jobs (permanently or temporarily) during the onset of the pandemic. In contrast, Dolado et al. (2021), using social security affiliation data for Spain, find that men experienced a greater reduction in employment than women during the first weeks of the lockdown, but that women were more likely to transit from employment into inactivity between the first and the second quarter of 2020.

**Table 1:** Gender differences in the impact of Covid-19 on labour market outcomes

	Employed			Inactive			Furlough (ERTE)		
	(1)	(2)	(3)	(4)	(5)	(6)	(7)	(8)	(9)
	All	No child	Child	All	No child	Child	All	No child	Child
Q2 $\times$ 2020	$-$0.043***	$-$0.047***	$-$0.035***	0.028***	0.032***	0.022***	0.144***	0.153***	0.133***
	(0.005)	(0.007)	(0.007)	(0.004)	(0.005)	(0.006)	(0.002)	(0.003)	(0.003)
Q3 $\times$ 2020	$-$0.022***	$-$0.031***	$-$0.005	0.002	0.005	$-$0.004	$-$0.004*	$-$0.005	$-$0.003
	(0.005)	(0.007)	(0.007)	(0.004)	(0.005)	(0.006)	(0.002)	(0.003)	(0.003)
Q4 $\times$ 2020	$-$0.015***	$-$0.026***	0.002	$-$0.002	$-$0.001	$-$0.004	$-$0.004*	$-$0.001	$-$0.007**
	(0.005)	(0.007)	(0.007)	(0.004)	(0.005)	(0.006)	(0.002)	(0.003)	(0.003)
Q1 $\times$ 2021	$-$0.022***	$-$0.021***	$-$0.019***	0.013***	0.009**	0.017***	0.019***	0.023***	0.014***
	(0.004)	(0.005)	(0.005)	(0.003)	(0.004)	(0.004)	(0.002)	(0.002)	(0.002)
Q2 $\times$ 2020 $\times$ Female	$-$0.009	$-$0.006	$-$0.014	0.019***	0.008	0.034***	0.017***	0.014***	0.022***
	(0.007)	(0.010)	(0.010)	(0.006)	(0.008)	(0.008)	(0.003)	(0.004)	(0.004)
Q3 $\times$ 2020 $\times$ Female	$-$0.009	$-$0.003	$-$0.021**	0.008	$-$0.004	0.023***	0.007**	0.004	0.011**
	(0.007)	(0.010)	(0.010)	(0.006)	(0.008)	(0.008)	(0.003)	(0.004)	(0.004)
Q4 $\times$ 2020 $\times$ Female	$-$0.012*	$-$0.000	$-$0.030***	0.003	$-$0.005	0.013	0.001	$-$0.001	0.005
	(0.007)	(0.010)	(0.010)	(0.006)	(0.008)	(0.008)	(0.003)	(0.004)	(0.004)
Q1 $\times$ 2021 $\times$ Female	0.010*	$-$0.001	0.018**	$-$0.015***	$-$0.003	$-$0.028***	0.006***	0.006*	0.007**
	(0.005)	(0.007)	(0.007)	(0.004)	(0.006)	(0.006)	(0.002)	(0.003)	(0.003)
Observations	533,417	295947	237,470	533,417	295,947	237,470	397,387	211,081	186,306

Source: Own calculations based on quarterly EPA microdata (Q1/2019–Q1/2021)

Significance levels: *0.10 **0.05 ***0.01. “All” refers to the sample of individuals aged 25–54. “No child” refers to the sub-sample of individuals who do not have a child aged 15 or below in the household. “Child” refers to the sub-sample of individuals who do have a child aged 15 or below in the household. “Employed” (columns 1–3) is a dummy variable equal to one if the individual is employed and zero if the individual is unemployed or inactive. “Inactive” (columns 4–6) is a dummy variable equal to one if the individual is inactive and zero if the individual is employed or unemployed. “Furlough (ERTE)” is a dummy variable equal to one if the individual did not work in the reference week due to ERE/ERTE or technical and economic reasons, and zero if the individual is employed and not on ERE/ERTE. Only coefficients of interest are shown from the differences-in-differences specifications defined in Eq. (1). The coefficients denoted $Q1 \times 2021$ and $Q1 \times 2021 \times Female$ refer to $\alpha _52{,}021$ and $\alpha _72{,}021$ of Eq. (1), respectively. All regressions use cross-sectional weights

We update the existing evidence on the gendered impact of the Covid-19 pandemic on labour market outcomes using data from the Spanish Labour Force Survey up until the first quarter of 2021, focusing on individuals of child-bearing age and comparing those with and without children aged 15 and below. To do so, we follow a differences-in-differences strategy that compares the evolution of three labour market outcomes for men and women, before the Covid-19 pandemic (all four quarters of 2019 and the first quarter of 2020) and during the Covid-19 pandemic (second quarter of 2020 to the first quarter of 2021). We estimate equations of the following form:

1 $$\begin{aligned} y_i= & {} \alpha _0 + \alpha _1 Fem_i + \sum _{j=2020}^{2021} \alpha _{2j} Year_j + \sum _{k=2}^{4} \alpha _{3k} Q_k + \sum _{j=2020}^{2021} \alpha _{4j} Year_j \times Fem_i \nonumber \\&+\,\sum _{k=2}^{4}\alpha _{5k} Q_k \times Year_{2020} + \sum _{k=2}^{4} \alpha _{6k} Q_k \times Fem_i \nonumber \\&+\,\sum _{k=2}^{4} \alpha _{7k} Q_k \times Year_{2020} \times Fem_i + \epsilon _i \end{aligned}$$

where the main outcomes of interest (*y*) are: (1) being employed, (2) being inactive and (3) being on furlough (ERTE)), measured at the individual level. The first two outcomes are defined on the sample of all individuals aged 25–54, while the latter outcome is defined on the sample of employed individuals aged 25–54. *Fem* is a dummy variable equal to one for females, $\sum _{j=2020}^{2021} Year_j$ is a set of dummy variables for the years 2020 to 2021 (the omitted year is 2019), and $\sum _{k=2}^{4} Q_k$ is a set of dummy variables for quarters two to four (the first quarter is omitted). $\epsilon _i$ is an individual level error term. The coefficients of interest are the triple interaction terms between the female dummies, quarters two to four and the year 2020 ($\sum _{k=2}^{4}\alpha _{7k}$), plus the interaction between the female dummy and the year 2021 ($\alpha _{4,2021}$). The former capture the average change in the outcome between the first and subsequent quarters of a given year for women, above and beyond changes between the first and subsequent quarters of 2019 and those experienced by men during quarters two to four in 2020. The latter captures the same effect during the first quarter of 2021, since the data only go until then.

Results are shown in Table 1. In the full sample of men and women aged 25-54, we do not observe a statistically significant difference in the impact of the pandemic on the likelihood of being employed (Column (1)) up to the third quarter of 2020, after which the gender gap turns in favour of men. By the fourth quarter of 2020, women experienced a drop in employment rates 1.2 percentage points higher than males when compared to the first quarter of 2019. Women’s inactivity rate increased relatively more than that of males, but only during the second quarter of 2020 (Column (4)). Women were slightly more likely to have been put on furlough (Column (7)) throughout the pandemic (except for the fourth quarter of 2020), with coefficients on the triple interactions ranging from 1.7 percentage points in the second quarter of 2020 to 0.6 percentage points in the first quarter of 2021.

**Fig. 8:**
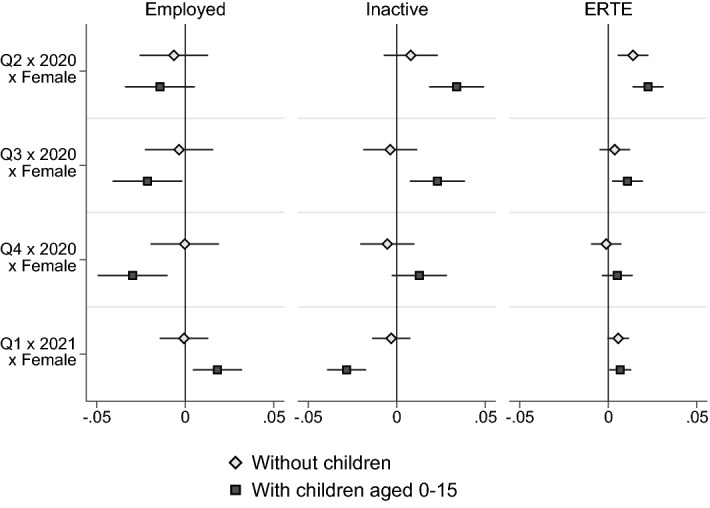
Gender differences in the impact of Covid-19 on labour market outcomes—by whether has children Own calculations based on EPA microdata from Q1/2019-Q1/2021. The graph shows the regression coefficients and their 95% confidence intervals from estimations of Equation (1), from separate regressions for the samples of individuals aged 25-54 with and without children aged zero to 15. “Employed” is a dummy variable equal to one if the individual is employed and zero if the individual is unemployed or inactive. “Inactive” is a dummy variable equal to one if the individual is inactive and zero if the individual is employed or unemployed. “ERTE” is a dummy variable equal to one if the individual did not work in the reference week due to ERE/ERTE or technical and economic reasons, and zero if the individual is employed and not on ERE/ERTE. Coefficients are shown in Table 1. The coefficient denoted $Q1 \times 2021 \times Female$ refers to $\alpha _4,2021$ of Equation (1).

Results are more mixed when looking at the sample of individuals with and without children separately. The coefficients on the triple interactions for these two sub-samples are plotted in Fig. 8. They show that among people without children, there are no discernible gender gaps, while they are clearly visible among people with children. Employment rates had dropped more among mothers than fathers between the second and the fourth quarter of 2020, and inactivity rates had risen more for mothers. Note that by the first quarter of 2021, the gender gap among individuals with children had reversed in terms of employment, which declined by 1.8 percentage points less for mothers than for fathers, and inactivity, which had fallen by 2.8 percentage points more than that of men. Mothers remained more likely to be on furlough than fathers by 0.7 percentage points. This reversal of the initially more negative impact of the pandemic on women is consistent with recent findings by Bluedorn et al. (2021), who show that in countries where women were initially hit harder (which experienced so-called she-cessions), this tended to last only one to two quarters.

While the pandemic shock might have been only temporary, evidence on employment interruptions for childcare shows that these can have long-lasting negative consequences for female earnings due to a loss in human capital (see, for instance, Schönberg and Ludsteck (2007)). Additionally, the unequal labour market impact of the Covid-19 pandemic for people with children might reinforce traditional gender roles. Time-use data collected by Farré et al. (2020) during the 2020 April and May lockdown in Spain shows that women were more likely to assume the main responsibility for most of housework and childcare, even when both parents were working. They estimate an increase in the gender gap in time dedicated to childcare by more than one hour per day during the pandemic. Similar increases in the gender gap in childcare have been found for other countries (e.g. in Hupkau and Petrongolo (2020) for the UK). The Covid-19 pandemic might therefore have long-term impacts on gender equality, both in the workplace and at home. It remains to be seen whether these trends persist once the pandemic is over, or whether dynamics born from the crisis are reversed.

## Family policies and their impact on gender equality

The fact that the arrival of children seems to be closely linked to the remaining gender gap in career outcomes raises the question of what can be done to reduce this “motherhood penalty”. Many countries have recently introduced changes to family policies with the explicit objective of reducing gender inequality related to childbirth. The first group of such policies are parental leave policies and the introduction of so-called daddy-quotas, which are non-transferable periods of parental leave reserved for fathers. Such policies are thought to encourage a more equal division of childcare within the household, thereby eroding traditional gender norms, facilitating women’s return to work after childbirth, and reducing bias by employers against women (Dunatchik and Özcan 2020).

Over the past 13 years, Spain has seen a drastic increase in the daddy-quota. While between 2007 and 2017 fathers only enjoyed two weeks of paid leave, between 2017 and 2021 this gradually increased to 16 weeks, equal to the amount of leave reserved for mothers. Increases in paternity leave have been introduced in other countries over the past decades (e.g. Sweden, Norway), sometimes coupled with explicit incentives for fathers to take up those benefits, such as an increase in the overall parental leave entitlement if both mother and father take up a sufficient amount of their individual leave entitlements (Germany).

The empirical evidence on the effects of these policies is somewhat mixed, but more recent studies point towards positive effects on women’s job outcomes. Farré and González (2019), for instance, show that the introduction of two weeks paternity leave in 2007 in Spain increased both mother’s likelihood of being in work six and 12 months after giving birth, as well as earnings in the two years following childbirth, facilitated by increased involvement of fathers in child rearing. Analysing the introduction of five weeks of non-transferable paternity leave in 2006 in Quebec (Canada), Dunatchik and Özcan (2020) show that it increased the labour force participation, the likelihood of working full-time, and decreased the likelihood of working part-time for mothers in the first three years after giving birth. The policy also increased father’s time spent on unpaid domestic work (Patnaik 2019). A number of other studies also find that increases in paternity leave lead to increased sharing of childcare duties within the household. For a policy introduced in Germany, granting additional leave if parents share some of the leave entitlement, Bünning (2015) finds that fathers increased the time spent with children and decreased the time spent working. This policy had positive effects on mothers’ employment rates, job continuity and job quality (Kluve and Schmitz 2018).

Another set of family policies has focused on financial assistance, consisting in either child subsidies or tax credits. With respect to the first kind of policy, González (2013) shows a negative short-run effect on maternal labour supply after the introduction of a one-off unconditional cash transfer of () 2,500 for new mothers in Spain. Magda et al. (2020) conclude that the introduction of a universal benefit for the second and every further child in Poland reduced mother’s labour market participation, especially among women with lower levels of education. Asakawa and Sasaki (2020) found that a reduction in the Japanese child benefit led to an increase in the participation rate of mothers of young children. This evidence suggests that the introduction of universal child subsidies has an income effect that negatively affects maternal labour supply. With respect to tax credits, or in-work benefits related to childbirth, Bastian and Lochner (2020), Bastian (2020) and Hoynes et al. (2015) show that the introduction or expansion of the Earned Income Tax Credit (EITC) in the USA had a positive effect on maternal employment. Shirley (2020) finds similar effects but that these are concentrated on unmarried women with low education.

The third set of policies concerns the cost of childcare. Lowering the cost of childcare or subsidizing it tends to have positive impacts on mother’s labour market attachment (Haeck et al. 2015; Bettendorf et al. 2015; Nollenberger and Rodríguez-Planas 2015; Bick 2016; Müller and Wrohlich 2020). However, the effect is heterogeneous in terms of magnitude depending on the country’s initial cost of childcare. When childcare costs are initially low, Givord and Marbot (2015) show that the introduction of a substantial childcare subsidy in France increased mothers’ labour force participation only marginally. Looking at cross-country evidence, an OECD (2018) report finds that higher levels of enrolment in early childhood education for children below the age of three are associated with higher rates of maternal employment. The report also shows that in countries where children aged zero to three spend more time in formal childcare settings, the share of part-time employment for working mothers is lower.

**Fig. 9:**
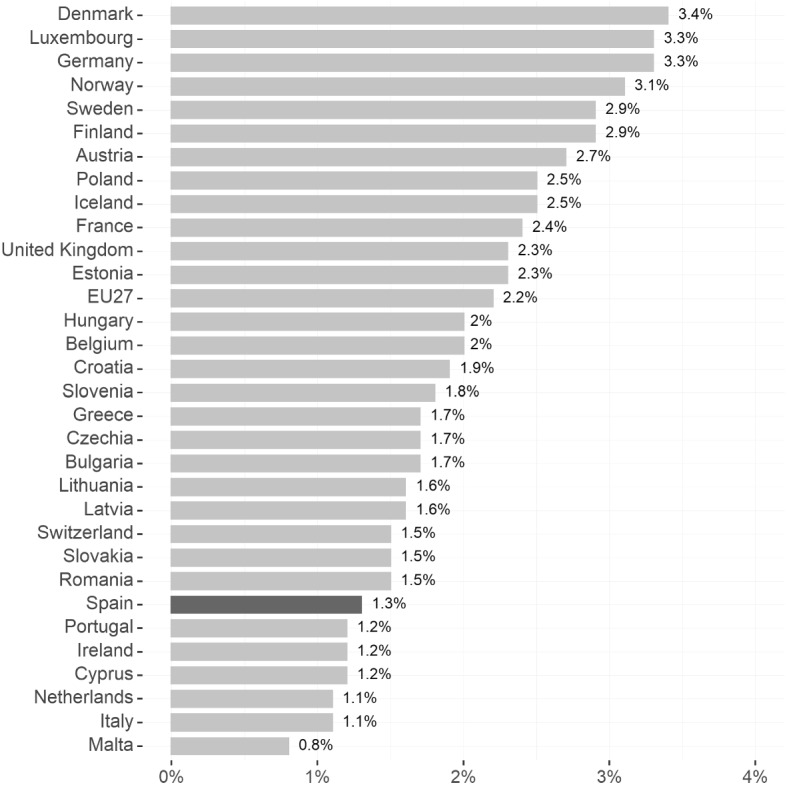
Expenditure on family or children benefits as percentage of GDP (2018) Source: Eurostat, Social Protection Expenditure. According to the European System of integrated Social Protection Statistics (ESSPROS) Manual and User guidelines the expenditure on family and children includes cash benefits (income maintenance benefit in the event of childbirth, birth grant, parental leave benefit, family or child allowance and other cash benefits ) and benefits in kind (child day care, accommodation, home help and other benefits in kind)

In the case of Spain, enrolment in early childhood education for children aged zero to three stood at 38 percent in 2014, close to the OECD average of 34 percent (OECD 2017). At the same time, public expenditure on family and child benefits is comparatively low. Figure 9 shows that Spain only spends 1.3 percent of GDP on such policies, while the EU27 average is 2.2 percent. Childcare costs for two children aged two and three years are estimated to represent about 25 percent of the average earnings of a two-earner household in Spain, which is close to the OECD average of just under 30 percent (OECD 2018). Both the existing evidence on the effect of lowering childcare costs on maternal labour market outcomes, and the current level of spending on these policies in Spain, suggest that there is potential for such policies to improve women’s participation and particularly to increase full-time employment rates.

**Fig. 10:**
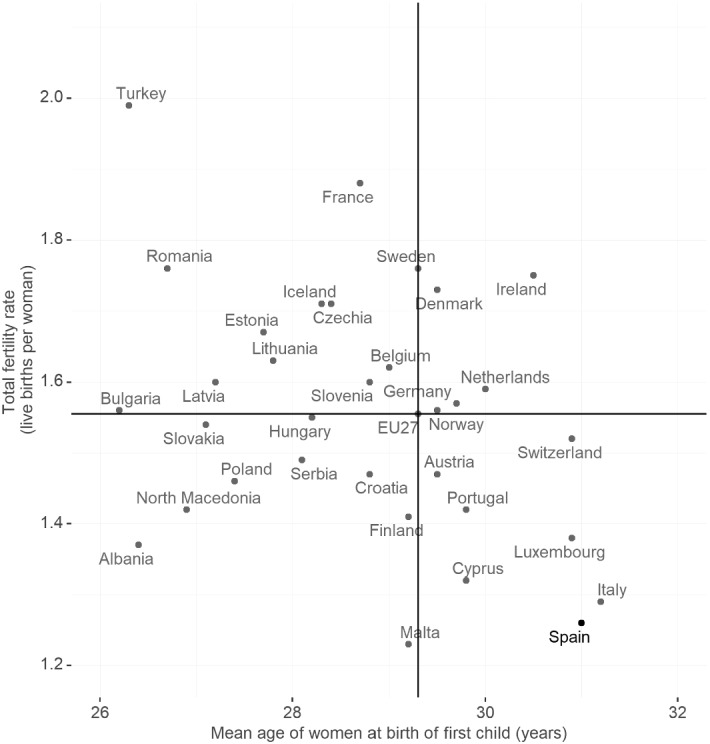
Fertility indicators across countries (2018) Source: Eurostat (2018). Reproduced from “Statistics explained: fertility indicators”, Fig. 4

In the face of declining fertility rates across most developed countries, family policies have also increasingly been used to incentivise child bearing. With only 1.3 live births per woman in 2018, Spain has among the lowest fertility across European countries (Fig. 10). At the same time, on average women have their first child at 31, compared to the EU-27 average of just over 29 years. Some of the policies reviewed above have been shown to positively affect women’s labour market outcomes, but they sometimes have negative effects on fertility (and vice-versa). On the one hand, the increase in paternity leave in Spain analysed in Farré and González (2019) caused a reduction in the likelihood of having further children. On the other hand, policies that negatively affect female labour force participation, like extensions of maternity leave or more generous child benefits, have been shown to increase fertility.

Table 2 summarises the most recent available evidence on the effects of different kinds of family policies on maternal employment and fertility. Overall, policies that make it easier to combine work and family for women, such as financial incentives in the form of tax credits for working mothers and subsidised or free childcare for very young children, have been shown to raise both women’s labour market attachment as well as fertility. Increased spending on such policies would thus likely reduce the motherhood penalty at the same time as raising fertility.

However, such policies alone are unlikely to be effective if not coupled with other reforms that address the organisation of work (Goldin 2014). Recent evidence by Guner et al. (2020) assesses the role of dual labour markets—with widespread employment insecurity due to high levels of temporary contracts—and inflexible work schedules in explaining low levels of fertility. They suggest that reforms enhancing work flexibility and reducing labour market duality would allow women to have children earlier, with the potential to increase fertility substantially.

Still, policies that only introduce the possibility of more flexible working conditions might exacerbate existing gender gaps. In general, women have been shown to choose jobs with family-friendly characteristics after the arrival of children. This includes flexible hours (Goldin 2014), being in the public sector or in lower level occupations (Kleven et al. 2019), offering higher levels of temporary flexibility (Azmat et al. 2021), or offering lower commuting times (Petrongolo and Ronchi 2020). All these job characteristics tend to be associated with lower pay. Thus, some family-friendly policies might actually reinforce gender inequality if they are only taken up by women, by fomenting employer’s beliefs about women’s comparative advantage in childcare and reinforcing traditional gender roles (Olivetti and Petrongolo 2016). A striking example is the introduction of a law in Spain granting employment protection to workers with young children who had asked for a shorter working week due to family responsibilities. Fernandez-Kranz and Rodriguez-Planas (2021) show that in response to the introduction of the law, employers were less likely to hire women of childbearing age, and less likely to promote them to permanent contracts.

Moreover, the effectiveness of family policies is likely to be a function of the strength of gendered preferences.^6^ In settings with strongly entrenched gender norms, even generous family policies are unlikely to make a big difference for gender equality. Berniell et al. (2020), for instance, show that the negative labour market responses to motherhood are larger in countries with more conservative social norms. Breaking up traditional gender norms thus seems crucial for enhancing equality in the labour market. Indeed, as we showed in Sect. 3, over a third of women with children under five and more than half of women with children aged five to 15 working part-time would like to work more hours. One in two women with very young children name childcare responsibilities as the main reason why they do not work more hours, while this is much less of a driver for part-time work among fathers.

The question is thus whether preferences can be steered towards more gender equal norms. There is some evidence that this is possible. Fernández et al. (2004), for instance, show that sons whose mothers worked are more likely to have working wives, and they attribute this to the fact that men who grew up in a family where the mother worked may be less averse to having a working wife. As pointed out above, the equalisation of parental leave length for Spanish parents might well induce a shift in gender norms towards a more equal division of housework and childcare.

## Conclusion

Over the past 25 years Spain has undergone a striking convergence between women’s and men’s participation in the labour market. By 2020, the Spanish labour market showed over 88 active women for every 100 active males, whereas in the early 1990s only 50 women were active in the labour market for every 100 men. This has meant that Spanish women’s labour market participation has overtaken the average in the European Union.

**Table 2:** Family policies and their impact on women’s job outcomes and fertility

Type of policy	Examples	Objective	Effect on maternal employment	Effect on fertility
Parental leave	Maternity leave	Increase flexibility	**Mixed evidence**	**Mixed evidence**
	Paternity leave	Increase fathers’ involvement in childcare	Short-run negative effect of increased maternity leave in Lalive et al. (2013), Schönberg and Ludsteck (2014), Bergemann and Riphahn (2015), Ginja et al. (2020)	Positive effect in Lalive and Zweimüller (2009), Malkova (2018), Raute (2019)
	Parental leave		In general, positive effects are found for short to moderate-sized entitlements (Olivetti and Petrongolo 2017), while longer job-protected leave periods can be detrimental. Long-run outcomes for mothers are likely unaffected by maternity leave policies (Kleven et al. 2020)	Negative effect in Farré and González (2019)
			Positive effect of increased parental leave in Farré and González (2019), Byker (2016), Dunatchik and Özcan (2020), Kluve and Schmitz (2018)	
Financial assistance	Child cash benefits	Boost birth rates	**Mixed evidence**	**Positive effect**
	Child tax credit/benefit	Promote employment and reduce poverty	Negative effect of increased cash benefits in González (2013), Magda et al. (2020)	González (2013), González and Trommlerová (2020), Sorvachev and Yakovlev (2020), Cohen et al. (2013), Bastian (2017)
			Positive effect of increased tax benefits in Hoynes et al. (2015), Bastian (2020), Bastian and Lochner (2020)	Not significant in Riphahn and Wiynck (2017)
			Not significant in Milligan and Stabile (2009)	
Availability of subsidised/free child-care	Public education 0–3 years old	Increase mothers’ labour market attachment	**Positive effect**	**Mixed evidence**
	Childcare voucher		Haeck et al. (2015), Bettendorf et al. (2015), Nollenberger and Rodríguez-Planas (2015), Müller and Wrohlich (2020)	Positive effect in Bauernschuster et al. (2016), Olivetti and Petrongolo (2017)
				Neutral effect in Nollenberger and Rodríguez-Planas (2015), Bick (2016)

Using data from the Spanish Labour Force Survey, we show that despite overall convergence between men and women in participation rates, women still fare worse on other important measures such as unemployment rates, the percent of women in temporary or part-time contracts and the percent of women holding positions in the top occupation (i.e. directors and managers). Not only do women fare worse, the gender gap in those measures has not improved over the past 15 years. This is despite the fact that the Great Recession from 2008 to 2013 disproportionately hit male-dominated sectors, and during these years gender gaps narrowed. However, once the economic growth resumed, the gender gap in these main indicators started rising once more.

The situation is further aggravated for women with children, irrespective of the indicator used. Convergence in labour force participation rates has stagnated for women with children aged 15 and below over the last seven years. By the end of the 2010s, women with children under 15 years of age were about 7.5 times more likely than men (with children of the same age) to be employed on part-time contracts, twice as likely to be unemployed and 20 percent more likely to hold a temporary contract. Part of these disparities could be driven by different preferences between men and women (and especially between mothers and fathers) regarding time use. However, the data indicate that it is unlikely that preferences alone can explain these differences in labour market attachment. Over a third of women with children under five and more than half of women with children aged five to 15 working part-time would like to work more hours. The Covid-19 pandemic, with its unequal effects in the labour market across genders, is already contributing to widening existing gender gaps among parents—at work and at home.

A review of the literature on family policies suggests that there is scope for well-designed policies to help narrow gender inequalities in the labour market. Existing evidence shows that more generous paternity leave entitlements reserved for fathers, which Spain has recently implemented, increase female labour force participation, employment and earnings. They have also been shown to increase men’s involvement in childcare and therefore have the potential to change gender norms.

Policies that make it easier to combine work and family for women, such as financial incentives in the form of tax credits for working mothers and subsidised or free childcare for very young children, have also been shown to positively affect women’s labour market attachment. These latter policies would also help tackle a related issue: Spain is among the countries with the lowest fertility rates and the highest age of women at first birth. At the same time, Spain spends only about half of the average country in the EU-27 on family and child benefits. Increased spending on such policies would thus likely reduce the motherhood penalty at the same time as increasing fertility rates.

The welfare effect of such policies will depend on whether the gains in terms of tax revenues and economic output from increased female labour supply outweigh the cost of providing more affordable childcare or in-work benefits. Existing evidence suggests that women substantially increase both labour force participation and working hours in response to family policies that either provide financial assistance through tax credits or make childcare more affordable. Even if not all of the increased expenditure on childcare provision is covered by the increases in income, social security and payroll taxes generated from increased maternal employment, this does not necessarily mean that such policies would be inefficient. They may well bring other benefits, such as increases in fertility and, in the case of provision of free or affordable, high-quality childcare, improvements in children’s educational outcomes in primary and secondary school.

While the above policies have been shown to be effective in reducing the negative labour market impact of motherhood, they are unlikely to be sufficient to close the remaining gender gap unless they are coupled with (1) other reforms addressing the organisation of work and (2) breaking up traditional gender norms.

## Footnotes

1 Throughout the document, ’without children’ is a simplification used for ease of reading. Individuals without children (for the purpose of this article) are those that do not have children aged zero to 15 living in the same household.

2 We exclude 2020 from the main analysis because the primary goal of this section is to look at long-term trends, which have been interrupted by the economic recession caused by the Covid-19 pandemic. We analyse the gendered labour market impact of the Covid-19 pandemic in Sect. 3. See Guner et al. (2014)) for the evolution of employment rates, as well as the fraction of part-time and temporary workers by gender from the early 1970s through 2013.

3 We use publicly available EPA data that include 1-digit occupational codes. Because occupational classifications changed in 2011 from CNO-98 to CNO-11 (INE 2012), and as there is no one-to-one mapping between 1-digit CNO-98 and 1-digit CNO-11, we can only show time consistent data on occupational shares from 2011 onwards.

4 We do not use the Spanish Labour Force Survey for this analysis as it does not include data on earnings or income in their publicly available version.

5 GDP is estimated to have slumped by 11 percent in 2020 (Eurostat 2021a), and unemployment has risen from 13.9 percent in the last quarter of 2019 to 16.2 percent in the last quarter of 2020 (Eurostat 2021b).

6 See for instance Kleven et al. (2020) for a discussion.
